# Evaluation and comparison of the effectiveness of kaleidoscope and virtual reality goggles to reduce dental anxiety in young children undergoing administration of local anesthesia.

**DOI:** 10.12688/f1000research.134041.3

**Published:** 2024-02-05

**Authors:** Himani Parakh, Nilima Thosar

**Affiliations:** 1 Department of Pediatric and Preventive Dentistry, Sharad Pawar Dental College and Hospital, Datta Meghe Institute of Higher Education and Research (Deemed to be University), Wardha, Maharashtra, 442001, India

**Keywords:** Kaleidoscope, Virtual Reality, Distraction, Local anaesthesia, Young children

## Abstract

Administration of local anesthesia via injection is the main reason for inducing anxiety in children and if not intervened it aggravates the anxiety in subsequent appointments. There are many approaches, including pharmacological and non-pharmacological methods that can be used to reduce children’s perception of pain and anxiety. A frequently used non-pharmacological behaviour management method to reduce anxiety and procedure pain is distraction. The rationale of distraction is to shift the attention to avert the brain from receiving unwanted stimulus which induce anxiousness. Many approaches including music, television, portable video games, virtual reality (VR) helmet, and virtual reality audio-visual eyewear, have been implemented so far. Kaleidoscope and virtual reality goggles may help as distraction techniques in reducing the levels of anxiety caused during administration of local anesthesia. By the aid of which the operator may be able to make the dental experience child friendly and ultimately provide quality dental care to anxious children. Non pharmacological measures of reducing pain are cost effective and best means that can be used while performing dental procedures. This is a research protocol for a study aimed to evaluate and compare the effectiveness of kaleidoscope and virtual reality goggles to reduce dental anxiety in young children.

## Introduction

Anticipation of pain during dental treatment is a frequently reported reason for dental anxiety and fear. Dental anxiety comprises of various physical, mental as well as social components. Anxiety has been defined as a “vague, unpleasant feeling accompanied by a premonition that something undesirable is about to happen”. ^1^ In the terms of Dental Fear and Anxiety (DFA), Dental anxiety is characterized as significant negative or unpleasant feeling about a dental office and dental procedures; whereas dental phobia is an irrational form of dental anxiety. ^2^ Dental anxiety is said to be a major contributor to poor oral health. Several studies have shown that the majority of significant triggers come from the sight, sound, and vibratory sensation of dental drills, and sensation of a local anaesthetic injection which is a major cause; subsequently dental fear is associated with past painful experiences and negative staff behaviour. ^3^ An anxious patient is in a continuous state of restlessness and unease. Children experiencing anxiety during local anesthesia injection gives rise to a significant concern which thus necessitates cognitive - behavioural psychological guidance along with the needed dental treatment. ^4^ There are many approaches, including pharmacological and non-pharmacological methods that can be used to reduce children’s perception of pain and anxiety. ^5^^,^^6^ Amongst which the procedures that focuses on cognitive or behavioural methods include practices such as listening to music, dreaming, hypnosis, use of virtual reality goggles, progressive muscle relaxation, and distraction through various interventional techniques. ^7^ Attention refers to focusing and processing information from our surroundings. While in anxious state it is important to shift the child’s attention away from the unpleasant stimuli. Distraction works on the principle of diverting the focus and blocking the reception of the unpleasant stimuli that causes anxiety.

Kaleidoscope is an instrument that shows an infinite number of fascinating geometric shapes in the form of a flower, repeating and reflecting images of coloured goggle fragments in the front section in a prism mirroring the inner surface. Increased blinking of the eyes is directly proportional to task difficulty, which in turn produces stress. In a relaxed situation, such as during kaleidoscope viewing, the number of blinks decreases and thus it controls the general physiological excitation. ^8^^,^^9^ Kaleidoscope is being increasingly recognized for its therapeutic and healing value in medical procedures but very few studies on it are available for its use in pediatric dentistry. A study by Aditya *et al.* was conducted on the pediatric dental population with a kaleidoscope and virtual reality and a positive reduction in VPT scores and pulse rate was reported. ^10^

Virtual Reality (VR) is a human–computer interface that enables the user to communicate with the computer-generated atmosphere with dynamism. VR actively involves children and is a potent tool in helping children to keep their attention away from frightening and painful procedures. It allows the children to perceive the environment as a safe where they can be free without any risk. ^10^^,^^11^ Atzori *et al.* (2017) found that VR technology reduced the mean “worst pain” from 3.80 to 2.20, hence indicating that VR can help effectively distract and engage children undergoing dental treatments. ^12^

Hence the focus of this study, according to the available literature, is to evaluate and compare the effectiveness of kaleidoscope and virtual reality goggles to reduce dental anxiety in young children.

### Protocol

The aim of this study is to evaluate and compare the effectiveness of kaleidoscope and virtual reality goggles to reduce dental anxiety in young children undergoing administration of local anesthesia *.*

Objectives are:

1. To evaluate the effect of kaleidoscope to reduce dental anxiety levels in young children before and after the administration of local anesthesia.
2. To evaluate the effect of virtual reality goggles to reduce dental anxiety levels in young children before and after the administration of local anesthesia.
3. To compare effect of kaleidoscope and virtual reality goggles technique to reduce dental anxiety levels in young children before and after the administration of local anesthesia.

This parallel randomized multiple-arm clinical trial will be conducted in an isolated setup over a period of six months. It will be a trial for equivalence. Children from five - eight years old who are healthy, co-operative but anxious. Children without any past dental experience those will undergo dental treatment requiring administration of local anesthesia for procedures like pulpectomy or extractions will be included in the study. Children with known vision problems, psychological abnormalities, children with special health care needs, children suffering from systemic diseases, and the parents that are unwilling to participate will be excluded from the study.

**Group 1 – Kaleidoscope:** The children will be given a kaleidoscope having various changing colours and patterns to watch through just before the starting of the procedure and will be asked to watch through it until the procedure ends.

**Group 2 – Virtual reality goggles technique:** The children will be asked to wear Virtual Reality goggles and a video to keep the child engaged will be played by clicking on the remote control. VR will be given just before the starting of the procedure and will be asked to watch through it until the procedure ends.

**Group 3 –** Control group wherein usage of basic behavior guidance techniques will be employed.

It is a randomized clinical trial. This is a version 1 of the protocol. Sequence generation by lottery method will be generated. The co-investigator of the department will generate the allocation sequence and will assign participants to interventions. It will be a double-blind study where trial participants, data analysts will be blinded. Data management by double data entry will be done. Personal information about potential and enrolled participants will be collected and maintained in order to protect confidentiality before, during, and after the trial. Comparators Kaleidoscope, Virtual reality goggles technique and conventional behaviour management method will be taken into consideration for comparing as all of them are interventions aiding for reducing the anxiety levels.

The sample size calculation is based on previous research by Aditya *et al.* ^13^ that used a similar study technique by using the sample size formula for difference between two means, $\frac{{\left(z\alpha +z\beta \right)}^{2}\left({\delta_{1}}^{2}+\delta_{2/k}^{2}\right)}{{∆}^{2}}$.

A total of 60 children will be included for the study. The participants in each group will be allocated by simple random sampling.

VFAS score using a validated Visual Facial Anxiety Scale is the primary outcomes to be measured. Pulse oximeter to measure physiological signs of oxygen saturation, and pulse rate, respiratory rate are the secondary outcomes to be measured. All the outcomes will be measured before, during, and after intervention.

### Data analysis and statistical plan

Analytical tests like Chi square test and student’s t-test will be performed. All the statistical analysis will be performed using SPSS software, version 27.0 p<0.05 will be considered as the level of significance. This randomized clinical trial will be conducted at the Department of Pediatric and Preventive Dentistry, Sharad Pawar Dental College, Datta Meghe Institute of Higher Education and Research, Wardha. The allocation will be done using lottery method.

### Dissemination

Once completed the study will be published in a PubMed, Scopus indexed journal.

## Discussion

There is a limited literature available regarding the use kaleidoscope as a distraction method for children undergoing dental procedures. Aditya *et al.* conducted a study to compare various methods of distraction to control anxiety in 60 children between the age group of six - nine years divided into four groups. Group 1 was fidget spinner (FS), Group 2 was kaleidoscope, Group 3 Virtual reality and Group 4 was control group with no distraction. The child’s self-reported anxiety levels using Venham’s picture test (VPT); the pulse oximeter was used to measure physiological signs of oxygen saturation and pulse rate at three intervals i.e. before, during, and after the IANB procedure. The VPT values were lower in the first three groups. Hence it was concluded that Fidget spinner, kaleidoscope, and virtual reality seem to be effective distraction methods and can be recommended as effective approaches to help reduce children’s dental anxiety during IANB procedures. ^13^ Tailor *et al.* found out that when Virtual reality (VR) and on-screen audio-visual distraction techniques were used in the age group of 4-8 years the VPT scores were significantly reduced in VR group as compared to On-screen aid group in the first two visits that included procedures of screening, cavity preparation and L.A. administration. ^14^ The effect of cartoon display and kaleidoscope in children between the ages of 5-12years was assessed and the anxiety levels were scored using Children’s Fear Scale by Molu and Açıkgöz, during echocardiography procedure. A significant difference is evident in anxiety scores before and during between all the intervention groups and it was concluded that use of kaleidoscope is an effective measure in reducing anxiety. ^15^ Distraction by Kaleidoscope and VR is a promising area for future research in pediatric dentistry because of the rising interest in non-pharmacological methods for behaviour management.

### Ethical considerations

Ethical approval for the study was obtained from the Institutional ethics committee of Datta Meghe Institute of Medical Sciences (Deemed to be University) [ref no: DMIMSU (DU)/IEC/2022/756].

The study protocol will be explained to the participating children and their parents.

Further, a written informed consent will be obtained from their parents.

Trial Registration is done under The *Clinical Trials Registry - India*, the registry number is (CTRI/2023/04/051258- REF/2023/03/065058)

Date of Registration: 03/04/2023

https://ctri.nic.in/Clinicaltrials/rmaindet.php?trialid=83188&EncHid=56660.93573&modid=1&compid=19

### Study status

Yet to be started.

## Data Availability

No data is associated with this article. Zenodo. SPIRIT checklist for “Evaluation and comparison of the effectiveness of kaleidoscope and virtual reality goggles to reduce dental anxiety in young children undergoing administration of local anesthesia”. DOI: https://doi.org/10.5281/zenodo.7811753
