# Supplementary material for: Evaluation and comparison of the effectiveness of kaleidoscope and virtual reality goggles to reduce dental anxiety in young children undergoing administration of local anesthesia
Source: F1000Res. 2024 Aug 21;12:546. Originally published 2023 May 24. [Version 4] doi: 10.12688/f1000research.134041.4 (PMC11344195; doi:10.12688/f1000research.134041.4)
Supplement: Peer review history

# Reviewer report: Reviewer response for version 4

**DOI:** 10.5256/f1000research.170260.r315779

**Published:** 2024-08-23

**Authors:** M. A. Jaber

**Affiliations:** 1 Ajman University, Ajman, United Arab Emirates

Thank you for sharing the revised manuscript with the changes. I have reviewed the modifications made by the authors in response to the concerns I raised in my peer-review report. Upon careful consideration, I believe that the authors have addressed the majority of the issues appropriately. The revisions have improved the clarity and robustness of the manuscript, and the key concerns have been adequately resolved.

 I am pleased to provide my updated approval status for this version.

Is the study design appropriate for the research question?

Partly

Is the rationale for, and objectives of, the study clearly described?

Yes

Are sufficient details of the methods provided to allow replication by others?

Partly

Are the datasets clearly presented in a useable and accessible format?

Yes

Reviewer Expertise:

Dental Education, Oral precancer, Oral Cancer, Salivary glands, facial injuries

I confirm that I have read this submission and believe that I have an appropriate level of expertise to confirm that it is of an acceptable scientific standard.

# Reviewer report: Reviewer response for version 3

**DOI:** 10.5256/f1000research.161946.r304492

**Published:** 2024-08-09

**Authors:** M. A. Jaber

**Affiliations:** 1 Ajman University, Ajman, United Arab Emirates

**1. Introduction**

 The study proposal aims to compare the effectiveness of a kaleidoscope and virtual reality goggles in reducing dental anxiety in children during local anesthesia administration. Dental anxiety is a significant issue affecting oral health, with various triggers and potential interventions mentioned in the proposal.

**2. Identification of Gap in Literature**

 The proposal successfully identifies a gap in the literature concerning the use of kaleidoscope as a distraction method in pediatric dentistry. However, the gap regarding VR goggles is less pronounced, as there is already substantial evidence supporting its effectiveness.

**3. Soundness and Rigor of the Proposal**

 Strengths:

- The study addresses a pertinent issue in pediatric dentistry.
- The use of kaleidoscope and VR goggles as non-pharmacological interventions is innovative and has potential therapeutic value.

Weaknesses:

- The literature review, while comprehensive, lacks critical analysis of the cited studies. It should delve deeper into the methodologies and limitations of the referenced research to build a stronger case for the proposed study.
- The proposal lacks a clear theoretical framework explaining why these particular interventions are chosen and how they are hypothesized to work in reducing dental anxiety.

**4. Methodology**

 Strengths:

- The use of a randomized, double-blind clinical trial design is robust and enhances the reliability of the results.
- The sample size calculation based on previous research is appropriate.

Weaknesses:

- The exclusion criteria are broad, potentially limiting the generalizability of the findings. For instance, excluding children with past dental experiences might omit a significant portion of the target population.
- The control group lacks a detailed description of the "basic behavior guidance techniques" to be employed, which could lead to inconsistencies in the control conditions.

**5. Reliability and Validity of the suggested methodology**

 Strengths:

- Double-blinding enhances the internal validity of the study.
- The use of VFAS scores and physiological measures (pulse oximeter readings) is appropriate for assessing anxiety levels.

Weaknesses:

- The proposal does not specify the exact timing of data collection (e.g., how long after the procedure the anxiety levels will be measured).
- The lottery method for randomization, while simple, may not be as effective in ensuring complete randomness compared to computerized randomization techniques.

**6. Suggestions for Improvement**

- Conduct a more in-depth analysis of existing studies, highlighting their methodologies, findings, and limitations.
- Develop a theoretical framework to explain the expected mechanisms through which kaleidoscope and VR goggles reduce dental anxiety.
- Provide a detailed protocol for the behavior guidance techniques used in the control group to ensure consistency.
- Reconsider the exclusion criteria to include children with past dental experiences to enhance generalizability.
- Consider using computerized randomization methods to ensure complete randomness and reduce potential biases.
- Specify the exact timing of anxiety measurements to standardize the data collection process.

**7. Overall assessment**

 This study proposal is well-structured and addresses an important issue in pediatric dentistry. While the methodology is robust, there are areas for improvement in the literature review, theoretical framework, and control group definition. By addressing these areas, the study can significantly contribute to the understanding and management of dental anxiety in children.

Is the study design appropriate for the research question?

Partly

Is the rationale for, and objectives of, the study clearly described?

Yes

Are sufficient details of the methods provided to allow replication by others?

Partly

Are the datasets clearly presented in a useable and accessible format?

Yes

Reviewer Expertise:

Dental Education, Oral precancer, Oral Cancer, Salivary glands, facial injuries

I confirm that I have read this submission and believe that I have an appropriate level of expertise to confirm that it is of an acceptable scientific standard, however I have significant reservations, as outlined above.

# Response

**Published:** 2024-08-10

**Authors:** Himani Parakh

**Affiliations:** Pedodontics, Datta Meghe Institute of Higher Education and Research, Wardha, Maharashtra, India

Respected sir,

 Thank you for your valuable insights and suggestions.

 1. Information about the mechanisms through which kaleidoscope and VR goggles reduce dental anxiety is mentioned in the introduction with regards to the regulation of the general physiological excitation and attention shift.

 2. By comparing Kaleidoscope to a well-established tool like VR, we aim to fill the existing research gap and provide a more comprehensive understanding of Kaleidoscope's potential in this area.

 3. I have made the corrections in the manuscript regarding the behavior guidance techniques used in the control group.

 4. The children with past dental experiences are excluded because the behaviors and responses to dental procedures are shaped by these experiences, which may influence the outcomes of our study.

 5. Due to limited access to specific software, for the due course of this study lottery method was assigned.

 6. The exact timing of anxiety measurements to standardize the data collection process is updated.

 I have uploaded version 4 with the modifications as quoted.

 Regards,

 Dr. Himani Parakh

# Reviewer report: Reviewer response for version 2

**DOI:** 10.5256/f1000research.160680.r237961

**Published:** 2024-01-27

**Authors:** Abhishek Lal

**Affiliations:** 1 Department of Medicine, The Aga Khan University, Karachi, Sindh, Pakistan

Comment 1: The eligibility criteria needs to be refined. The patients included were those with first time dental experience or with previous dental experiences.

 Comment 2: Please provide the references of the few studies as stated by the authors which have been conducted similar to this research in the introduction section.

 Comment 3: Is there any reasons as to why only 5-8 years of participants were recruited?

 Comment 4: Are all the operating procedures standardized for all patients regardless of which group they belong to?

 Comment 5: Please provide information regarding the blinding process followed in this study.

Is the study design appropriate for the research question?

Yes

Is the rationale for, and objectives of, the study clearly described?

Yes

Are sufficient details of the methods provided to allow replication by others?

Partly

Are the datasets clearly presented in a useable and accessible format?

Not applicable

Reviewer Expertise:

Public Health Dentistry, Oral Health, Oral Pathology, Mental Health, Non-communicable diseases.

I confirm that I have read this submission and believe that I have an appropriate level of expertise to confirm that it is of an acceptable scientific standard, however I have significant reservations, as outlined above.

# Response

**Published:** 2024-01-27

**Authors:** Himani Parakh

**Affiliations:** Pedodontics, Datta Meghe Institute of Higher Education and Research, Wardha, Maharashtra, India

Respected sir,

 Thank you for your valuable suggestions.

 1: I have made the corrections in the manuscript regarding the inclusion of patients with first-time dental experience.

 2: References of the few studies that have been conducted similar to this research are added in the introduction section and quoted in the discussion section too.

 3: 5 to 8 years age participants were recruited because, in this age group, children in this age range are anxious in a dental setup owing to their emotional and psychological development.

 4: The operating procedures are standardized by limiting it to injecting block anesthesia for either pulpectomy or extraction procedures for all patients regardless of which group they belong to.

 5. Information about blinding is modified as a double-blind study in the protocol section of the manuscript.

 I have uploaded version 3 with all the modifications as quoted.

 Regards,

 Dr. Himani Parakh

# Reviewer report: Reviewer response for version 1

**DOI:** 10.5256/f1000research.147067.r197357

**Published:** 2023-09-15

**Authors:** Rekhalakshmi Kamatham, Dileep Nag Vinnakota

**Affiliations:** 1 Department of Pediatric and Preventive Dentistry,, Narayana Dental College and Hospital, Nellore, Andhra Pradesh, India; 2 Department of Prosthodontics, Narayana Dental College, Nellore, Andhra Pradesh, India

It is an interesting protocol. The literature on the comparison of kaleidoscope and virtual reality to reduce dental anxiety in young children is meager. However, there are certain flaws in the methodology:

- Authors failed to mention the type of randomized clinical trial and the trial design; whether it is superiority, inferiority or equivalence trial; whether it is parallel or split mouth.
- Can elaborate on the inclusion criteria like the procedures that the authors want to consider.
- Control group intervention should be modified to usage of basic behavior guidance techniques. We feel it is not proper to mention no such distraction technique. 
- Authors can consider other validated outcome measures to assess the anxiety in the children of the considered age group. Please mention whether the visual facial anxiety scale is validated or not. Should clearly mention the primary and secondary outcome measures. 
- The sample size determination is not correct. What is the study that the authors have considered for sample size determination?
- The authors have wrongly mentioned simple random sampling method. It should not be a sampling method!! Need to modify the randomization, sequence generation methods.
- What about blinding? Authors did not mention about blinding aspect.
- What is the use of Chi square test? Please describe in detail the application of each test.

Is the study design appropriate for the research question?

Yes

Is the rationale for, and objectives of, the study clearly described?

Yes

Are sufficient details of the methods provided to allow replication by others?

Partly

Are the datasets clearly presented in a useable and accessible format?

Not applicable

Reviewer Expertise:

Pediatric dentistry- Behavioral management; Biostatistics

We confirm that we have read this submission and believe that we have an appropriate level of expertise to confirm that it is of an acceptable scientific standard, however we have significant reservations, as outlined above.

# Response

**Published:** 2023-12-26

**Authors:** Himani Parakh

**Affiliations:** Pedodontics, Datta Meghe Institute of Higher Education and Research, Wardha, Maharashtra, India

Thank you for your valuable suggestions.

 I have made the corrections in the manuscript regarding the type of randomization, refinement of the inclusion criteria, and outcome measures. Sample size calculation based on key articles is updated.

 Information about blinding is available in the protocol section of the manuscript.

 To test the null hypothesis, I would like to add that the Chi-square test will be applied as the sample size is more than 30. 

 I have uploaded the version 2 with all the modifications as quoted.
